# Genomic Profiling and Molecular Characterization of Clear Cell Renal Cell Carcinoma

**DOI:** 10.3390/curroncol30100670

**Published:** 2023-10-20

**Authors:** Gaetano Pezzicoli, Federica Ciciriello, Vittoria Musci, Francesco Salonne, Anna Ragno, Mimma Rizzo

**Affiliations:** 1 Department of Interdisciplinary Medicine, University of Bari “Aldo Moro”, 70124 Bari, Italy; g.pezzicoli@studenti.uniba.it (G.P.); f.ciciriello3@studenti.uniba.it (F.C.); v.musci4@studenti.uniba.it (V.M.); francesco.salonne@uniba.it (F.S.); 2 Medical Oncology Unit, Azienda Ospedaliera Universitaria Consorziale, Policlinico di Bari, 70124 Bari, Italy; annavincenza.ragno@policlinico.ba.it

**Keywords:** renal cell carcinoma, genomic profiling, VHL, mTOR, chromatin modulators, DNA repair genes, molecular tumor board, liquid biopsy

## Abstract

Clear cell renal cell carcinoma (ccRCC) treatment has undergone three major paradigm shifts in recent years, first with the introduction of molecular targeted therapies, then with immune checkpoint inhibitors, and, more recently, with immune-based combinations. However, to date, molecular predictors of response to targeted agents have not been identified for ccRCC. The WHO 2022 classification of renal neoplasms introduced the molecularly defined RCC class, which is a first step in the direction of a better molecular profiling of RCC. We reviewed the literature data on known genomic alterations of clinical interest in ccRCC, discussing their prognostic and predictive role. In particular, we explored the role of VHL, mTOR, chromatin modulators, DNA repair genes, cyclin-dependent kinases, and tumor mutation burden. RCC is a tumor whose pivotal genomic alterations have pleiotropic effects, and the interplay of these effects determines the tumor phenotype and its clinical behavior. Therefore, it is difficult to find a single genomic predictive factor, but it is more likely to identify a signature of gene alterations that could impact prognosis and response to specific treatment. To accomplish this task, the interpolation of large amounts of clinical and genomic data is needed. Nevertheless, genomic profiling has the potential to change real-world clinical practice settings.

## 1. Introduction

Renal cell carcinoma (RCC) is the most common renal neoplasm [1]. Several environmental factors, including cigarette smoking, obesity, hypertension, and alcohol consumption, contribute to the development of RCC [2]. In 5–8% of cases, a hereditary condition is detectable [3]. The clear cell histotype (ccRCC) is the most frequent, representing 75% of the whole population, while the other cases are grouped as non-clear cell RCC (nccRCC), which is a wide definition, comprising many different histologies [4]. The five-year survival rate is 13% in patients with metastatic disease [1].

The clinical management of metastatic ccRCC radically changed over the last thirty years. Starting from cytokines (high-dose interleukin 2 and interferon-α), with poor results, the systemic treatment of metastatic ccRCC evolved with the introduction of vascular endothelial growth factor receptor tyrosine kinase inhibitors (VEGFR-TKIs), mechanistic target of rapamycin inhibitors (mTORIs), and immune checkpoint inhibitors (ICIs). This led to a measurable improvement: the median overall survival for metastatic disease has increased from less than 1 year in the 1990s to more than 4 years over the last decade [5]. Different immune-based combinations (i.e., combinations of either two ICIs, or of one ICI and an antiangiogenic agent) recently showed significant OS benefits over the previous standard of care. However, most patients with advanced ccRCC do not have a long-term clinical response to currently available therapies. In addition, the combinations of ICIs and VEGFR-TKIs reduce the overall treatment tolerability, due to cumulative adverse events, which, in some cases, can even reinforce each other (e.g., immune-mediated colitis and VEGFR-TKI-induced diarrhea or ICI-induced myocarditis and VEGFR-TKI-mediated microvascular damage) [6]. Moreover, given the recent unsuccessful clinical trials performed to boost first-line and subsequent line treatments, no major therapeutic change is expected in the near future. Hence, now more than ever, there is a need for better profiling of ccRCC patients, which could enable the development of more specific drugs and thus lead to pursuing a fully agnostic therapeutic approach.

The trend toward a profiling-based approach is already evident in the most recent classification of urogenital tumors (WHO 2022) [7], which identifies a total of 21 different forms of RCC, including a new category called “molecularly-defined RCC”. This category includes TFE3-rearranged RCC, TFEB-rearranged RCC, TFEB-amplified RCC, FH-deficient RCC, SDH-deficient RCC, ALK-rearranged RCC, ELOC (formerly TCEB1)-mutated RCC, and SMARCB1 (INI1)-deficient RCC. The molecular data underpinning these entities seem to be relevant in the differential diagnosis from a pathological point of view; however, their predictive value is much more limited [8]. This is due to the intrinsic complexity of ccRCC alterations: while many other tumors rely on few strong oncogenic drivers, such as oncogene-addicted non-small cell lung cancer or BRAFV600E-mutated melanoma, ccRCC has a wide range of mutations and many of them concur to boost angiogenesis. This effect can be directly elicited, by the mutated protein per se acting as an enhancer of angiogenic pathways, or indirectly obtained, by the mutated proteins exerting epigenetic manipulation. The question becomes even more complex if we consider the strong spatial and temporal heterogeneity of ccRCC [9]. As a consequence, ccRCC follows its own set of rules when it comes to response to treatments. These considerations could, on the one hand, explain the shortcomings in the identification of viable druggable targets and, on the other hand, reinforce the preclinical and clinical evidence that angiogenesis continues to play a key pathogenic role during the whole natural history of ccRCC [10].

The present paper aims to review the most recent literature to depict a clear landscape of mutational signatures in ccRCC, highlighting the possibility of clinical exploitation.

## 2. Molecular Alterations of Clinical Interest in ccRCC

Clear cell RCC accounts for more than two thirds of all RCC cases. Therefore, it is the most studied histotype of RCC. Many data on the correlation between genomic alterations and treatment response come from clinical trials that included only RCC with a predominantly clear cell component. The most studied genes are VHL and some chromatin remodeling modulator genes located on the short arm of chromosome 3, close to VHL.

### 2.1. VHL (Von Hippel Lindau Syndrome Protein) and Its Network

**Figure 1:**
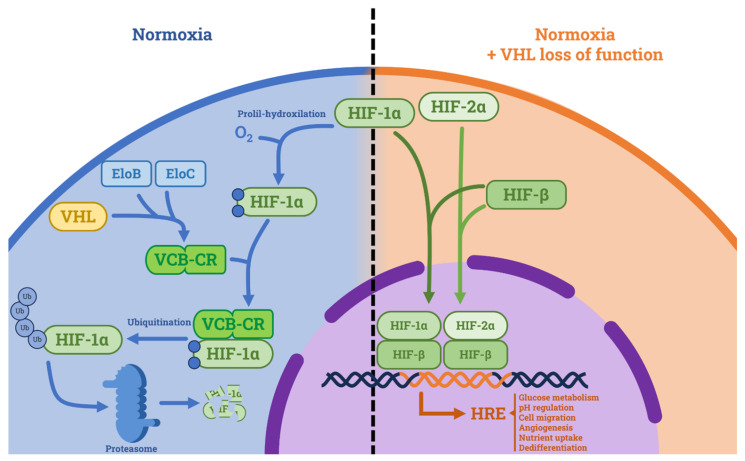
Tumor suppressive role of VHL and consequences of its loss of function. In normoxic conditions, VHL forms a complex with Elongin C and Elongin B (namely VCB-CR) and induces proteasome-mediated HIF degradation. In hypoxic conditions and in VHL loss of function, HIF is not degraded. HIF-1α and HIF-2α form complexes with HIF-β and translocate in the nucleus where they activate the hypoxia-related elements (HREs), which cause tumorigenic effects. Abbreviations: HIF: hypoxia inducible factor, HRE: hypoxia-related element, EloB: Elongin B, EloC: Elongin C, VCB-CR: VHL-Elongin C-Elongin B-Cullin 2-Ring Box 1 complex.

Von Hippel Lindau syndrome is an autosomal dominant genetic disorder caused by a germline loss of function of VHL, resulting in increased susceptibility to several types of cancer, mainly ccRCC [11]. VHL mutations have also been demonstrated in 46–82% of all sporadic cases of ccRCC, making it the most commonly altered oncogene in this type of tumor [12]. VHL’s role in ccRCC carcinogenesis is pivotal: the first event of this process is a chromothripsis event, leading to rearrangement and subsequential loss of the 3p chromosome and gain of the 5q chromosome [13]. VHL loss of function causes impaired ubiquitylation and removal of hypoxia inducible factors (HIFs). Accumulated HIFs trigger the production of many growth factors and the stimulation of angiogenesis (Figure 1) [14]. More recent data on tumor heterogeneity highlighted that ccRCC has both cells with a VHL loss-of-function mutation, and cells that retain VHL function. The interplay between VHL+ and VHL- cells results in increased tumor aggressiveness, whereas tumors with only VHL- or VHL+ cells have minimal metastatic potential [15].

The prognostic role of VHL is limited. A cohort of 478 ccRCC patients from The Cancer Genome Atlas (TCGA) showed no correlation between clinical outcomes and VHL mutations [16]. The prognostic value of VHL mutations in ccRCC has been the object of many other studies over the last two decades. A meta-analysis including 633 patients confirmed that VHL gene alteration has no prognostic or predictive value in patients with ccRCC [17].

However, something might change with the introduction of the HIF-2α inhibitor Belzutifan (MK-6482) into clinical practice. Belzutifan acts by selectively and directly inhibiting HIF-2α, the member of the HIF family with the highest oncogenic potential, thus being particularly active in cells with VHL loss. The phase II LITESPARK-004 trial enrolled patients with germline VHL mutations, including 61 patients with localized RCC, and demonstrated an objective response rate (ORR) of 64% [18]. Enrolment in the ongoing trials, LITESPARK-005 (NCT04195750), LITESPARK-011 (NCT04586231), and LITESPARK-012 (NCT04736706), needs to be completed to assess whether VHL loss could be a predictive factor of response to Belzutifan in ccRCC since Belzutifan is administered regardless of the VHL status in the aforementioned trials. Table 1 shows an overview of clinical trials involving Belzutifan.

Based on these results, VHL should be considered a gene with limited prognostic value, but with a promising efficacy prediction role for the HIF-2α inhibitor Belzutifan (MK-6482).

### 2.2. Mechanistic Target of Rapamycin (mTOR, Previously Known as Mammalian Target of Rapamycin) and Its Network

The second most studied molecular mechanism involved in ccRCC is the PI3K/AKT/mTOR signaling pathway. Mutations in this axis promote the overactivation of the PI3K/AKT/mTOR signaling cascade [19] or the inhibition of the tumor suppressor PTEN. PTEN, PI3K, AKT, and mTOR represent key checkpoints of the signal transduction pathway involved in the control of cell development and proliferation [20,21]. In fact, alterations in the PI3K/AKT/mTOR pathway can be detected in 28% of clear cell RCC cases. Preclinical data highlight that mTOR mutations are particularly represented in highly vascularized areas of the tumor since vascularization allows nutrients and growth factors to activate the mTOR pathway [22].

The prognostic role of mTOR network mutations is debated [23]. Fan et al. demonstrated that the increased expression of mTOR and its related proteins correlates with better survival [24]. On the other hand, Ocana et al. reported that mutations of the mTOR axis, in particular the loss of PTEN, correlate with a significantly worse prognosis [25]. The predictive role of mTOR network mutations is even more difficult to define: a report by Voss et al. [26] highlighted that the vast majority of patients with long-lasting responses to mTOR inhibitors harbor genomic alterations with an activating effect on mTOR signaling. Similarly, Roldan-Romero et al. [27] reported that mTOR pathway mutations and PTEN loss were significant predictors of partial responses to mTOR inhibitors. However, there is only one prospective clinical trial evaluating this aspect [28]. In this histology-agnostic trial, a pan-cancer cohort of patients with mTOR mutations was treated with Everolimus, obtaining a disappointing ORR of 7%. Thus, although mTOR mutations could play a role in predicting response to Everolimus, it is likely that they are not the only factor in the equation. It is worth noting that Voss and Roldan-Romero’s investigations were not limited only to mTOR mutations but also analyzed mutations of its network.

Based on these findings, while mTOR mutations are not clearly linked to prognosis, they could predict the magnitude and duration of objective response to mTOR inhibitors.

### 2.3. Chromatin Remodeling Modulators

**Figure 2:**
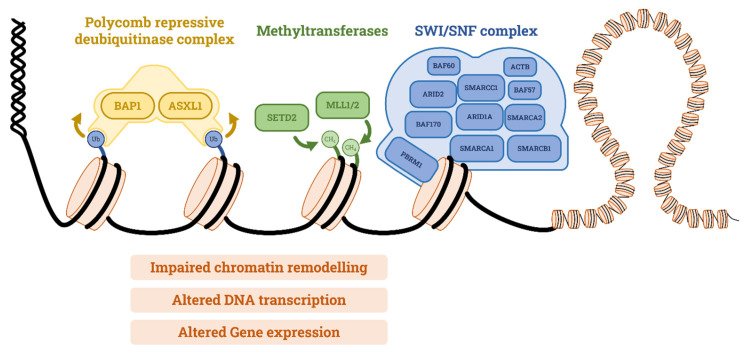
Main chromatin remodeling systems involved in RCC tumorigenesis: Polycomb repressive deubiquitinase complex involves BAP1 and acts by deubiquitinating histones. Methyltrasferases, such as SETD2, are enzymes that add a methyl group to histones. SWI/SNF complex is a molecular machinery that acts by sliding nucleosomes, ejecting nucleosomes, and ejecting only certain nucleosome components. All these systems modulate access to DNA transcription and, therefore, gene expression. Abbreviations: BAP1: BRCA-associated protein 1, ASXL1: putative Polycomb group protein ASXL1, SETD2: SET domain containing 2, MLL1/2: myeloid/lymphoid or mixed-lineage leukemia 1 and 2, SWI/SNF: SWItch/sucrose non-fermentable, PBRM1: Polybromo 1, SMARC: SWI/SNF-related matrix-associated actin-dependent regulator of chromatin, ARID: AT-rich interactive domain-containing protein, BAF: BRG1-associated factor.

Chromatin remodeling is an epigenetic regulation system, the magnitude and ability of which are not yet fully understood. Altered chromatin remodeling leads to rewired DNA transcription and impaired DNA damage repair. In ccRCC, altered chromatin remodeling is a consequence of 3p chromosome loss, which contains the chromatin remodeling modulator genes PRBM1, SETD2, and BAP1 (Figure 2). As 3p chromosome loss is an early event in ccRCC, as aforementioned, these alterations are quite common.

#### 2.3.1. PBRM1

Polybromo 1 is a protein encoded by PBRM1 and is involved in the PBAF complex (an SWI/SNF chromatin remodeling complex) known for its tumor suppressor role. PBAF, in fact, acts by regulating cellular proliferation and differentiation [29,30]. PBRM1 mutations are very common in ccRCC, and their frequency ranges between 30% and 40% of patients in the most referenced case series [16,31]. PBRM1 is a close neighbor of VHL in the 3p chromosome, so its loss is frequently associated [32]. However, its loss has a more important impact on prognosis. Specifically, in a case series of 132 patients with ccRCC, PBRM1 loss showed an association with longer relapse-free survival [33]. The predictive role of PBRM1 was also investigated. A 2016 study of 31 patients with metastatic ccRCC treated with a VEGFR-targeting agent found that patients that experienced a duration of response longer than 21 months had a significantly higher frequency of PBRM1 mutations, while this effect was not observed with mTOR inhibitors [34]. A similar study reported a higher frequency of durable objective response among patients with PBRM1 mutations [35]. PBRM1 status was also evaluated in some prospective clinical trials, with controversial results: In the phase III RECORD-3 trial (Sunitinib vs. Everolimus as first-line treatment in patients with mccRCC) PBRM1-mutated patients had a longer PFS with Everolimus, while this effect was not observed in the Sunitinib arm [36]. In the phase III COMPARZ trial (Sunitinib vs. Pazopanib as first-line treatment in patients with mccRCC), overall survival was significantly better in patients with PBRM1 mutations compared with patients without PBRM1 mutations [37]. The phase III IMmotion150 trial (Sunitinib vs. Atezolizumab vs. Atezolizumab plus Bevacizumab as first-line treatment in patients with mccRCC), with its important molecular analysis design, offered a clearer view: the PBRM1-mutated tumors had a strong proangiogenic transcriptomic signature. Interestingly, patients with PBRM1 mutation had better progression-free survival in the Sunitinib arm and the Atezolizumab plus Bevacizumab arm, but not in the Atezolizumab monotherapy arm [38]. The impact of PBRM1 mutation on immunotherapy response remains difficult to understand: a recent pan-cancer analysis of 168 PBRM1-mutated tumors vs. 1743 nonmutated tumors revealed that in ccRCC the PRBM1 mutation has no association with immunotherapy response or immune-related biomarkers, while in other cancer types (endometrial carcinoma, stomach adenocarcinoma, and colon adenocarcinoma) this mutation is significantly associated with immune-related biomarkers [39]. On the other hand, another large-scale analysis describes a positive predictive role of SWI/SNF genes (including PBRM1) for ccRCC patients undergoing immunotherapy with immune checkpoint inhibitors [40]. A recent analysis pointed out that the combination of PBRM1 status and tumor-infiltrating lymphocyte density allows us to obtain a trustworthy prognostic model for ccRCC [41].

Accordingly, PBRM1 is a favorable prognostic factor and a potential positive predictive factor for immunotherapy.

#### 2.3.2. BAP1

BRCA-associated protein 1 (BAP1) is a tumor suppressor, with histone deubiquitinase activity which contributes to the formation of the Polycomb repressive complex PR-DUB, a molecular machinery involved in chromatin rearrangement and inhibition of cell proliferation [42]. Interestingly, BAP1 is a double-hit gene, meaning that both alleles have to lose function to impact cell phenotype [43]. In fact, BAP1 germline mutation is correlated with an increased incidence of RCC and other cancers (including uveal melanoma, malignant pleural mesothelioma, and cutaneous melanoma) [44]. Like PBRM1, BAP1 is located on the 3p chromosome, so its loss is frequently associated with the loss of PBRM1 and VHL. BAP1 mutations are present in 5–15% of ccRCC cases [16,33,45,46], and have been shown to be associated with metastatic disease at diagnosis, high Fuhrman grade, and presence of necrosis (the last two factors being clinical predictors of disease recurrence in localized disease) [47,48]. Furthermore, BAP1 has been demonstrated to increase the risk of disease recurrence in patients with resected localized disease: in a cohort of 1479 patients, those with BAP1 loss of function (10.3%) had worse relapse-free survival, even after statistical adjustment for the UISS risk score [49]. It is therefore expected that BAP1-mutated tumors show a shorter overall survival than non-BAP1-mutated tumors [50].

The relationship between BAP1 mutations and PBRM1 mutations has been extensively described. In a report by Peña-Llopis et al., BAP1 and PBRM1 mutations seem to be almost always mutually exclusive. The two genes activate two separate and distinct transcriptional programs, with BAP1 being associated with a higher tumor grade and a greater sensitivity to genotoxic stress, much more than PBRM1. Interestingly, in a small percentage of patients, BAP1 and PBRM1 mutations may coexist and in these cases, the resulting neoplasm manifests rhabdoid features [51]. These results were also confirmed in an analysis performed on the TCGA and UTSW databases by Kapur et al. showing a much longer OS for PBRM1-mutated patients (10.6 years in UTSW and 5.4 years in TCGA) than for BAP1-mutated patients (4.6 years in UTSW and 1.9 years in TCGA) [48]. The rare tumors with both mutations (found in the TCGA cohort) showed an OS of 0.2 years.

As for its possible predictive role, BAP1 mutations were addressed in the genomic analyses of the RECORD-3 and COMPARZ trials. In both studies, BAP1 loss of function was associated with a shorter PFS in patients treated with antiangiogenic drugs [37]. When it comes to immunotherapy, there is much preclinical evidence that BAP1 is associated with an immunosuppressive microenvironment: it has been demonstrated that BAP1 mutation is associated with T-cell suppression in preclinical RCC models [52]. Clinical data on BAP1 as a predictor of response to immunotherapy have only recently been published. In an analysis by Liu et al. based on the TCGA cohort and the genomic data from the JAVELIN Renal 101 trial (Avelumab plus Axitinib vs. Sunitinib as first-line in mRCC) and Checkmate-009/010/025 trials (Nivolumab vs. Everolimus in mRCC), BAP1-mutated tumors resulted in a longer estimated PFS with ICI therapies than with antiangiogenic/mTORi therapies (HR 0.71, 95% CI 0.51–0.99, *p* = 0.045) [53]. Moreover, they developed a BAP1 score, based on how many BAP1-mutation-associated genes were expressed. The ICI benefit over other treatments is significant in BAP1-mutated patients with a high BAP1 score (Avelumab plus Axitinib vs. Sunitinib: HR 0.55, 95% CI 0.43–0.70, *p* < 0.001; Nivolumab vs. Everolimus: HR 0.72, 95% CI 0.52–1.00, *p* = 0.045), while it is negligible for BAP1-mutated patients with a low BAP1 score. Therefore, the BAP1 mutation is not a predictor of ICI response per se, but rather an indicator of a gene expression signature that favors ICI use. This could easily be explained by the wide genomic-regulator effect that BAP1 exerts as a chromatin regulator.

To summarize, BAP1 mutations have a negative impact on prognosis and a high level of BAP1 expression could predict ICI therapy benefits.

#### 2.3.3. SETD2

Along with PBRM1 and BAP1, the SETD2 gene is located in the 3p chromosome. It is a histone-lysine N-methyltransferase that plays a role in transcription regulation and alternative splicing [54]. Its loss-of-function mutation has been observed to cause microsatellite instability [55,56]. Interestingly, its mutation is frequently associated with PBRM1 mutation, but the association of the two mutations has no greater impact on overall prognosis than the single PBRM1 mutation [57]. Its mutation is reported with a frequency of 13–30% in different case series [54]. SETD2 loss of function is usually subclonal, meaning that it is only present in some cells within a single tumor [58]. Its effect on prognosis is negative: it has been associated with a shorter time to disease recurrence after nephrectomy. On the other hand, when considering OS from the start of systemic treatment, SETD2-mutated patients do not differ significantly from SETD2 wild-type patients, as described in a retrospective analysis of 240 patients whose tumors were studied with whole-exome sequencing [59]. The authors suggest that this might be due to a greater frequency of BAP1 mutations in SETD2 wild-type patients.

As for the predictive role, SETD2 status was evaluated in the COMPARZ and RECORD-3 prospective clinical trials, but no significant association was found [37]. However, in two large pan-cancer retrospective studies, which enrolled 451 and 375 SETD2-mutated tumors, respectively, SETD2 loss of function correlated with a higher tumor mutation burden and a better response to ICI immunotherapy [60,61].

Ultimately, SETD2 should be evaluated for its negative impact on prognosis.

### 2.4. DNA Damage Repair (DDR) Genes

There are at least 450 genes that exert a DDR function, and they are often organized in DDR systems [62]. Among all the DDR systems, the most studied are base excision repair (BER, which includes OGG1 and XRCC1), nucleotide excision repair (NER, which includes ERCC1 and XP), mismatch repair (MMR, which includes MSH2 and MLH1), homologous recombination repair (HHR, which includes BRCA1 and BRCA2), and non-homologous end joining (NHEJ, which includes KU70 and KU80). DDR genes also include regulators that act as cell-cycle checkpoints, such as ATM and ATR.

A total of 27–32% of RCC cases harbor mutations in DDR genes [63]. However, their potential as predictive biomarkers is still debated. Impairment in DDR genes often leads to an increase in random mutations that cannot be repaired. Consequently, the neoantigen load increases, thus rendering DDR-altered tumors more immunogenic [64]. A predictive role of DDR gene mutations in patients undergoing ICI therapy has been described for colorectal cancer and bladder cancer [65,66].

As for ccRCC, a retrospective analysis of 229 patients compared ICI and VEGFR-TKI responses among DDR-altered and non-DDR-altered tumors. No difference in ICI PFS was documented, but patients with DDR alterations treated with ICIs showed a significantly longer OS [67]. Another retrospective study found that among ccRCC patients, DDR alterations were more common in those achieving disease control [68].

Other drugs of potential clinical interest are poly-ADP ribose polymerase inhibitors (PARPi). PARPi act by suppressing a BER system and causing the cancer cell to accumulate more and more mutations until this condition is incompatible with survival. PARPi have already shown great efficacy in DDR-altered tumors. Moreover, they showed efficacy in RCC preclinical models, both in vitro and in vivo [63]. A phase II clinical trial of Olaparib (a PARPi) in monotherapy in patients with DDR-altered RCC is currently ongoing and is expected to be completed by 2025 (NCT03786796).

Based on the above, the potential predictive role of DDR gene alterations has yet to be defined.

### 2.5. Cyclin-Dependent Kinases (CDKs)

CDKs are regulatory molecules necessary for the ordered progression of the cell cycle and function in the integration of cellular signals to control cell-cycle checkpoints [69]. They act by creating complexes with cyclin proteins at certain points in the cell cycle [70]. CDK4 and the closely related CDK6 are both dependent on D-type cyclins, whose expression increases in early G1 in response to various mitogenic stimuli [71]. These two molecules have been extensively studied and their inhibitors are currently used in clinical practice in the management of solid tumors (mainly breast cancer). The role of CDK4 and CDK6 in metabolism might be crucial in RCC, due to their interplay with many hypoxia-enhanced pathways and mTOR signaling [72].

Mutation, hypermethylation, or deletion of CDKN2A are correlated with decreased survival in many RCC subtypes [16]. Moreover, partial deletion of chromosome 9p, containing CDKN2A, is a frequent event specifically in ccRCC (among 20% in the TCGA cohort) [72]; results from an integrated proteogenomic analysis by the Clinical Proteomic Tumor Analysis Consortium showed that loss of chromosome 9p is associated with an upregulation of mTOR signaling effectors [73].

Regarding sensitivity to CDK 4/6 inhibitors, a preclinical study showed that, following Palbociclib treatment, RCC cells underwent G0/G1 cell-cycle arrest and late apoptosis, and loss of CDKN2A, CDKN2B, and E2F1 was significantly associated with Palbociclib sensitivity [74]. Different degrees of Ribociclib sensitivity have also been shown in different RCC cell lines [75]. Currently, the only clinical experience of CDK4/6 inhibitors in RCC is a phase I trial evaluating the combination of Abemaciclib and Sunitinib in metastatic RCC (NCT03905889).

Finally, CDK mutations are prognostically unfavorable and their potential predictive role is currently being evaluated.

### 2.6. Tumor Mutation Burden (TMB)

Albeit not a genomic alteration in the classical definition, TMB can be an important indicator and can have a predictive role. TMB is defined as the total number of single nucleotide variants and small insertions and deletions per one million bases (Mb) in a tumor tissue sample. TMB is commonly calculated using whole genome sequencing (WGS) or whole-exome sequencing (WES), but it can also be estimated with various targeted gene panels. On average, ccRCC has a TMB of 1.1 mutations per Mb [76].

Among solid tumors, a high TMB (as observed in melanoma, non-small cell lung cancer, and urothelial cancer) is associated with improved response to ICIs [77]. Conversely, low-TMB tumors do not usually respond to ICIs. Classically, this has been explained as a consequence of a larger number of neoantigens in tumors with higher TMB, which triggers immune response easily [78].

ccRCC, however, has a relatively low TMB, ten times lower than that of melanoma, but has shown good responses to ICIs. At the present time, ICIs represent an almost mandatory step in the treatment of a patient with ccRCC, both in monotherapy and in combination with VEGF-TKI. This could be due to an intrinsic limit of TMB and to the genomic alteration landscape of ccRCC: In this tumor, large insertions and deletions—which per definition do not account for the TMB—are common, and frequently cause a frameshift, thus creating large amounts of neoantigens. This causes a high neoantigenic load with a relatively low TMB [79].

The correlation between higher TMB and better overall survival in patients treated with ICIs can also be seen within the ccRCC cohort. In a recent analysis, improved survival was described for tumors with a TMB in the top 20% of the population (5.9 mutations/Mb in RCC) [77]. In the same population, TMB showed no predictive or prognostic role in patients who did not receive ICIs.

In conclusion, only a relatively high TMB can be considered a positive prognostic factor, but its predictive role remains limited.

## 3. Discussion and Future Perspectives

The role of genomic profiling of ccRCC is gaining more and more relevance in recent years due to advances in molecular comprehension. The latest WHO classification of RCC takes into account these considerations and, for the first time, it defines a category of molecularly defined RCC [8]. Our analysis focuses on ccRCC due to its higher incidence and the high volume and quality of the literature data.

In the genomic landscape of ccRCC, there are many molecular alterations of potential clinical interest. VHL is a commonly mutated gene in ccRCC, and its mutation causes a shift to a hypoxic and proangiogenic phenotype. Albeit its prognostic and predictive role is limited in the current clinical practice, something could change with the introduction of HIF-2α inhibitors, such as Belzutifan. Belzutifan has proven its efficacy on VHL syndrome patients, which are not the primary object of our report, since we focused on sporadic ccRCC due to its higher incidence and its unmet clinical needs. Belzutifan still has to prove its efficacy in sporadic ccRCC, whose genomic landscape is different from that of hereditary ccRCC. Other common alterations in sporadic ccRCC are those that affect mTOR and its network, leading to impaired control of cell development and proliferation. These mutations do not have a clear prognostic role, but some evidence suggests that they could predict response to mTORi. Then there are chromatin remodeling modulators, such as PBRM1, SETD2, and BAP1, whose alterations do not have predictive roles, but can significantly impact prognosis or time to disease recurrence after nephrectomy. Moving to less consolidated evidence, it is worth mentioning DNA repair gene mutations and cyclin-dependent kinase mutations, which could predict responses to PARP inhibitors and CDK inhibitors, respectively. Trials addressing this subject are currently ongoing. Finally, albeit not a single genomic alteration in a strict sense, TMB has shown some predictive role for ICI response.

However, when translating these data to clinical practice, some limitations must be considered. The first one is tumor heterogeneity. On one hand, there is interpatient heterogeneity: as discussed above, all the genomic alterations analyzed in this paper are present only in a part of the whole ccRCC population. On the other hand, there is intrapatient heterogeneity: it has been proven that in ccRCC the concordance between the primary tumor and metastases is not 100%, especially when we consider chromatin remodeling modulator mutations [80]. The question is even more complicated if we take into account intratumor heterogeneity, which is the difference between different areas of the same neoplastic mass. This has been demonstrated to be impactful for many genomic alterations in ccRCC [81]. The high heterogeneity of ccRCC does not allow us to easily develop a one-size-fits-all strategy. One possible strategy to overcome tumor heterogeneity is assessing the mutation status of the largest possible number of cancer cells from different tumor sites with an accessible and reproducible technique. Liquid biopsy could be the technique most suitable for this objective. Liquid biopsy consists in the collection of tumor biological materials from bodily fluids, principally peripheral blood. It has recently been demonstrated that the analysis of circulating tumor DNA, circulating tumor cells, extracellular vesicles, urinary and blood microRNA, and specific proteins is feasible and accurate in ccRCC, in both prognostic and diagnostic settings [82,83]. Liquid biopsy is not limited to one or few sites like classic biopsy, and therefore, it can give us information on the genomic and proteomic status of the whole disease, including all metastatic sites. Moreover, the inaccessibility of genomic testing in clinical practice has recently been overcome: a large NGS panel is available in most major hospitals, and in smaller hospitals, remote testing options can be adopted.

In addition, many key genomic alterations of ccRCC happen in complex control proteins (such as the chromatin remodeling modulators) and the description of all their effects is difficult to achieve. Therefore, it is highly unrealistic that the personalized approach to ccRCC will be like the approach to NSCLC; while in NSCLC a mutated driver exerts a specific oncogenic action and its blockade has a strong therapeutic effect, in ccRCC the altered proteins exert a myriad of micro-effects that combine with each other, and their integration determines the cancer cell growth and survival. For this reason, the only way to develop a clinical-genomic integration tool that could guide therapeutic choices is to genomically evaluate larger and larger populations. The analysis of this gargantuan mass of data could help to elaborate a model that explains how little every single mutation impacts the cancer phenotype and how much more important is the combination of more mutations, namely a gene signature. These findings could be applied on a larger scale, in order to build even more accurate prognostic and predictive models. The use of artificial intelligence will be pivotal to this aim, and its reliability has recently been proven with the development of a prognostic model for ccRCC based on purely clinical factors [84]. The integration of these models with molecular data will be the next step. Furthermore, the easy accessibility of large NGS panels in clinical practice will allow even more data to be generated, integrating results from prospective clinical trials and insights from retrospective real-world analyses [85].

Real-world data on clinical-genomic correlations in ccRCC are pivotal at this point, since much of the data described in the literature come either from whole-exome sequencing experiences, such as the TCGA cohort [16], or from genomic analyses performed during randomized clinical trials. This introduces some biases. First of all, many cohorts of ccRCC patients undergoing whole-exome sequencing include mainly patients with localized diseases, which is a selection bias; this can be interesting in terms of elaborating tools to predict disease recurrence, but it makes it difficult to have a clear idea of the mutational landscape of metastatic disease. In the case of clinical trials with a wide plan of molecular analyses, such as IMmotion150 [38], the selection bias is given by the trial’s inclusion/exclusion criteria themselves: only eligible patients are subjected to genomic analysis, thus leaving a part of the real-world population unstudied.

The ever-changing paradigm of modern oncology requires a shift in the medical oncologist mindset: if up until now a pathology report and a complete radiological staging were essential for therapy choice, in the future the knowledge of mutational landscape will be just as pivotal. In this scenario, the integration of different skills from different professionals could be the winning strategy; the development of molecular tumor boards including molecular pathologists, biologists, bioinformaticians, geneticists, medical oncologists, and pharmacists should be strongly encouraged. Molecular tumor boards could be the key to bringing genomic profiling into every-day practice since they could offer to many patients access to valid molecular profiling and could help the medical oncologist to interpret the results and choose the most personalized therapy for each individual. Moreover, the growing accessibility of genomic profiling will lead to an increase in clinical-genomic data production, which will lead to an easier validation of integrated prognostic models.

## 4. Conclusions

Genomic profiling of ccRCC is promising, although still difficult to apply in large-scale clinical practice, due to the limitations described in our review: (I) tumor heterogeneity, (II) limited accessibility of genomic profiling tests, and (III) lack of genomic data validation. To overcome these limiting factors, we have outlined and promoted some realistic strategies: (I) tumor heterogeneity could be addressed through increased and recurrent use of liquid biopsy; (II) adoption of remote genomic testing could solve the inaccessibility of genomic testing for smaller hospitals, and the establishment of molecular tumor boards in larger hospitals could ensure adequate resource allocation and appropriate use of genomic profiling; (III) the integration of ccRCC clinical-genomic data from prospective randomized trials and real-life case series could contribute to the validation of genomic data.

## Figures and Tables

**Table 1:** Principal clinical trials involving Belzutifan, a HIF-2α inhibitor.

Trial Name	Phase	Patients	Study Arms	Outcomes
MK-6482-01	I	43 advanced ccRCC pretreated patients (dose escalation cohort)	Single arm: Belzutifan monotherapy	Safety endpoint met; mPFS 14.5 mts; ORR 25%
MK-6482-03	II	52 advanced ccRCC pretreated (≤2 lines) patients (cohort 2)	Single arm: Belzutifan + Cabozatninib	mPFS 13.8 mts; ORR 30.8%; mDOR 18.6 mts; mOS (24.1 mts)
MK-6482-04	II	61 naïve VHL-mutated patients with localized RCC	Single arm: Belzutifan monotherapy	24 mts-PFS 96%; ORR 51%; mDOR NR; mOS NR
MK-6482-05	III	736 advanced ccRCC pretreated (≤3 lines, at least 1 ICI and VEGFR-TKI) patients	Belzutifan vs. Everolimus	Estimated completion: 2025
MK-6482-010	I	52 advanced ccRCC pretreated (≤1 line) patients	Single arm: Belzutifan monotherapy dose escalation	Estimated completion: 2025
MK-6482-011	III	706 advanced ccRCC pretreated (≤2 lines, at least 1 ICI) patients	Belzutifan + Lenvatinib vs. Cabozantinib	Estimated completion: 2024
MK-6482-012	III	1652 advanced ccRCC naïve patients	Belzutifan + Pembrolizumab + Lenvatinib vs. MK-1308A + Lenvatinib vs. Pembrolizumab + Lenvatinib	Estimated completion: 2026
MK-6482-013	II	154 advanced ccRCC pretreated (≤3 lines, at least 1 ICI) patients	Single arm: Belzutifan monotherapy dose escalation	Estimated completion: 2025
MK-6482-022	III	1600 radically resected ccRCC patients	Pembrolizumab + Belzutifan vs. Pembrolizumab (1-year treatment)	Estimated completion: 2027
MK-6482-024	II	180 advanced ccRCC pretreated (≤3 lines, at least 1 ICI) patients	Belzutifan + Palbociclib vs. Belzutifan	Estimated completion: 2027

Abbreviations: mPFS = median progression-free survival; ORR = objective response rate; mDOR = median duration of response; mOS = median overall survival; 24mts-PFS = 24 months progression-free survival.

## References

[1] Kidney and Renal Pelvis Cancer—Cancer Stat Facts. https://seer.cancer.gov/statfacts/html/kidrp.html.

[2] Santoni M., Piva F., Porta C., Bracarda S., Heng D.Y., Matrana M.R., Grande E., Mollica V., Aurilio G., Rizzo M. (2020). Artificial Neural Networks as a Way to Predict Future Kidney Cancer Incidence in the United States. Clin. Genitourin. Cancer.

[3] Nguyen K.A., Syed J.S., Espenschied C.R., LaDuca H., Bhagat A.M., Suarez-Sarmiento A., O’Rourke T.K., Brierley K.L., Hofstatter E.W., Shuch B. (2017). Advances in the diagnosis of hereditary kidney cancer: Initial results of a multigene panel test. Cancer.

[4] Chevrier S., Levine J.H., Zanotelli V.R.T., Silina K., Schulz D., Bacac M., Ries C.H., Ailles L., Jewett M.A.S., Moch H. (2017). An Immune Atlas of Clear Cell Renal Cell Carcinoma. Cell.

[5] Demasure S., Spriet I., Debruyne P.R., Laenen A., Wynendaele W., Baldewijns M., Dumez H., Clement P.M., Wildiers H., Schöffski P. (2021). Overall survival improvement in patients with metastatic clear-cell renal cell carcinoma between 2000 and 2020: A retrospective cohort study. Acta Oncol..

[6] Crocetto F., Ferro M., Buonerba C., Bardi L., Dolce P., Scafuri L., Mirto B.F., Verde A., Sciarra A., Barone B. (2023). Comparing cardiovascular adverse events in cancer patients: A meta-analysis of combination therapy with angiogenesis inhibitors and immune checkpoint inhibitors versus angiogenesis inhibitors alone. Crit. Rev. Oncol. Hematol..

[7] Moch H., Amin M.B., Berney D.M., Compérat E.M., Gill A.J., Hartmann A., Menon S., Raspollini M.R., Rubin M.A., Srigley J.R. (2022). The 2022 World Health Organization Classification of Tumours of the Urinary System and Male Genital Organs—Part A: Renal, Penile, and Testicular Tumours. Eur. Urol..

[8] Rizzo M., Caliò A., Brunelli M., Pezzicoli G., Ganini C., Martignoni G., Porta C. (2023). Clinico-pathological implications of the 2022 WHO Renal Cell Carcinoma classification. Cancer Treat. Rev..

[9] Golkaram M., Kuo F., Gupta S., Carlo M.I., Salmans M.L., Vijayaraghavan R., Tang C., Makarov V., Rappold P., Blum K.A. (2022). Spatiotemporal evolution of the clear cell renal cell carcinoma microenvironment links intra-tumoral heterogeneity to immune escape. Genome Med..

[10] Canino C., Perrone L., Bosco E., Saltalamacchia G., Mosca A., Rizzo M., Porta C. (2019). Targeting angiogenesis in metastatic renal cell carcinoma. Expert Rev. Anticancer. Ther..

[11] Kim W.Y., Kaelin W.G. (2004). Role of *VHL* Gene Mutation in Human Cancer. J. Clin. Oncol..

[12] Cowey C.L., Rathmell W.K. (2009). VHL gene mutations in renal cell carcinoma: Role as a biomarker of disease outcome and drug efficacy. Curr. Oncol. Rep..

[13] Mitchell T.J., Turajlic S., Rowan A., Nicol D., Farmery J.H., O’brien T., Martincorena I., Tarpey P., Angelopoulos N., Yates L.R. (2018). Timing the Landmark Events in the Evolution of Clear Cell Renal Cell Cancer: TRACERx Renal. Cell.

[14] Wiesener M.S., Münchenhagen P.M., Berger I., Morgan N.V., Roigas J., Schwiertz A., Jürgensen J.S., Maxwell P.H., Löning S.A., Frei U. (2001). Constitutive activation of hypoxia-inducible genes related to overex-pression of hypoxia-inducible factor-1alpha in clear cell renal carcinomas. Cancer Res..

[15] Hu J., Tan P., Ishihara M., Bayley N.A., Schokrpur S., Reynoso J.G., Zhang Y., Lim R.J., Dumitras C., Yang L. (2023). Tumor heterogeneity in VHL drives metastasis in clear cell renal cell carcinoma. Signal Transduct. Target. Ther..

[16] Ricketts C.J., De Cubas A.A., Fan H., Smith C.C., Lang M., Reznik E., Bowlby R., Gibb E.A., Akbani R., Beroukhim R. (2018). The Cancer Genome Atlas Comprehensive Molecular Characterization of Renal Cell Carcinoma. Cell Rep..

[17] Kim B.J., Kim J.H., Kim H.S., Zang D.Y. (2017). Prognostic and predictive value of VHL gene alteration in renal cell carcinoma: A meta-analysis and review. Oncotarget.

[18] ESMO 2022: Belzutifan, a HIF-2α Inhibitor, for von Hippel-Lindau (VHL) Disease–Associated Neoplasms: 36 Months of Follow-Up of the Phase 2 LITESPARK-004 Study. https://www.urotoday.com/conference-highlights/esmo-2022/esmo-2022-kidney-cancer/139495-esmo-2022-belzutifan-a-hif-2-inhibitor-for-von-hippel-lindau-vhl-disease-associated-neoplasms-36-months-of-follow-up-of-the-phase-2-litespark-004-study.html.

[19] Sato T., Nakashima A., Guo L., Coffman K., Tamanoi F. (2010). Single amino-acid changes that confer constitutive activation of mTOR are discovered in human cancer. Oncogene.

[20] Ma L., Teruya-Feldstein J., Behrendt N., Chen Z., Noda T., Hino O., Cordon-Cardo C., Pandolfi P.P. (2005). Genetic analysis of Pten and Tsc2 functional interactions in the mouse reveals asymmetrical haploinsufficiency in tumor suppression. Genes Dev..

[21] Manning B.D., Logsdon M.N., Lipovsky A.I., Abbott D., Kwiatkowski D.J., Cantley L.C. (2005). Feedback inhibition of Akt signaling limits the growth of tumors lacking *Tsc2*. Genes Dev..

[22] Hager M., Haufe H., Lusuardi L., Schmeller N., Kolbitsch C. (2011). PTEN, pAKT, and pmTOR Expression and Subcellular Distribution in Primary Renal Cell Carcinomas and Their Metastases. Cancer Investig..

[23] Pezzicoli G., Filoni E., Gernone A., Cosmai L., Rizzo M., Porta C. (2021). Playing the Devil’s Advocate: Should We Give a Second Chance to mTOR Inhibition in Renal Clear Cell Carcinoma?—Ie Strategies to Revert Resistance to mTOR Inhibitors. Cancer Manag. Res..

[24] Fan D., Liu Q., Wu F., Liu N., Qu H., Yuan Y., Li Y., Gao H., Ge J., Xu Y. (2020). Prognostic significance of PI3K/AKT/mTOR signaling pathway members in clear cell renal cell carcinoma. PeerJ.

[25] Ocana A., Vera-Badillo F., Al-Mubarak M., Templeton A.J., Corrales-Sanchez V., Diez-Gonzalez L., Cuenca-Lopez M.D., Seruga B., Pandiella A., Amir E. (2014). Activation of the PI3K/mTOR/AKT Pathway and Survival in Solid Tumors: Systematic Review and Meta-Analysis. PLoS ONE.

[26] Voss M.H., Hakimi A.A., Pham C.G., Brannon A.R., Chen Y.-B., Cunha L.F., Akin O., Liu H., Takeda S., Scott S.N. (2014). Tumor Genetic Analyses of Patients with Metastatic Renal Cell Carcinoma and Extended Benefit from mTOR Inhibitor Therapy. Clin. Cancer Res..

[27] Roldan-Romero J.M., Beuselinck B., Santos M., Rodriguez-Moreno J.F., Lanillos J., Calsina B., Gutierrez A., Tang K., Lainez N., Puente J. (2019). PTEN expression and mutations in *TSC1*, *TSC2* and *MTOR* are associated with response to rapalogs in patients with renal cell carcinoma. Int. J. Cancer.

[28] Adib E., Klonowska K., Giannikou K., Do K.T., Pruitt-Thompson S., Bhushan K., Milstein M.I., Hedglin J., Kargus K.E., Sholl L.M. (2021). Phase II Clinical Trial of Everolimus in a Pan-Cancer Cohort of Patients with mTOR Pathway Alterations. Clin. Cancer Res..

[29] Kenneth N.S., Mudie S., van Uden P., Rocha S. (2009). SWI/SNF Regulates the Cellular Response to Hypoxia. J. Biol. Chem..

[30] Hodges H.C., Kirkland J.G., Crabtree G.R. (2016). The Many Roles of BAF (mSWI/SNF) and PBAF Complexes in Cancer. Cold Spring Harb. Perspect. Med..

[31] Espana-Agusti J., Warren A., Chew S.K., Adams D.J., Matakidou A. (2017). Loss of PBRM1 rescues VHL dependent replication stress to promote renal carcinogenesis. Nat. Commun..

[32] Gao W., Li W., Xiao T., Liu X.S., Kaelin W.G. (2017). Inactivation of the PBRM1 tumor suppressor gene amplifies the HIF-response in VHL−/−clear cell renal carcinoma. Proc. Natl. Acad. Sci. USA.

[33] Gossage L., Murtaza M., Slatter A.F., Lichtenstein C.P., Warren A., Haynes B., Marass F., Roberts I., Shanahan S.J., Claas A. (2013). Clinical and pathological impact of *VHL, PBRM1, BAP1, SETD2, KDM6A*, and *JARID1c* in clear cell renal cell carcinoma. Genes, Chromosom. Cancer.

[34] Ho T.H., Choueiri T.K., Wang K., Karam J.A., Chalmers Z., Frampton G., Elvin J.A., Johnson A., Liu X., Lin Y. (2016). Correlation Between Molecular Subclassifications of Clear Cell Renal Cell Carcinoma and Targeted Therapy Response. Eur. Urol. Focus.

[35] Fay A.P., de Velasco G., Ho T.H., Van Allen E.M., Murray B., Albiges L., Signoretti S., Hakimi A.A., Stanton M.L., Bellmunt J. (2016). Whole-Exome Sequencing in Two Extreme Phenotypes of Response to VEGF-Targeted Therapies in Patients With Metastatic Clear Cell Renal Cell Carcinoma. J. Natl. Compr. Cancer Netw..

[36] Hsieh J.J., Chen D., Wang P.I., Marker M., Redzematovic A., Chen Y.-B., Selcuklu S.D., Weinhold N., Bouvier N., Huberman K.H. (2016). Genomic Biomarkers of a Randomized Trial Comparing First-line Everolimus and Sunitinib in Patients with Metastatic Renal Cell Carcinoma. Eur. Urol..

[37] Voss M.H., Reising A., Cheng Y., Patel P., Marker M., Kuo F., Chan T.A., Choueiri T.K., Hsieh J.J., Hakimi A.A. (2018). Genomically annotated risk model for advanced renal-cell carcinoma: A retrospective cohort study. Lancet Oncol..

[38] McDermott D.F., Huseni M.A., Atkins M.B., Motzer R.J., Rini B.I., Escudier B., Fong L., Joseph R.W., Pal S.K., Reeves J.A. (2018). Clinical activity and molecular correlates of response to atezolizumab alone or in combination with bevacizumab versus sunitinib in renal cell carcinoma. Nat. Med..

[39] He X., Xin Y., Yuan H., Tao H., Wang Q., Zhu H. (2022). Pan-cancer analysis of *PBRM1* mutation and their association with immune-related biomarkers and prognosis. J. Clin. Oncol..

[40] Alaiwi S.A., Nassar A., El Bakouny Z., Berchuck J.E., Nuzzo P., Flippot R., Flaifel A., Steinharter J.A., Baca S., Margolis C. (2019). Association of polybromo-associated BAF (PBAF) complex mutations with overall survival (OS) in cancer patients (pts) treated with checkpoint inhibitors (ICIs). J. Clin. Oncol..

[41] Deutsch J.S., Lipson E.J., Danilova L., Topalian S.L., Jedrych J., Baraban E., Ged Y., Singla N., Choueiri T.K., Gupta S. (2023). Combinatorial biomarker for predicting outcomes to anti-PD-1 therapy in patients with metastatic clear cell renal cell carcinoma. Cell Rep. Med..

[42] Scheuermann J.C., de Ayala Alonso A.G., Oktaba K., Ly-Hartig N., McGinty R.K., Fraterman S., Wilm M., Muir T.W., Müller J. (2010). Histone H2A deubiquitinase activity of the Polycomb repressive complex PR-DUB. Nature.

[43] Carbone M., Harbour J.W., Brugarolas J., Bononi A., Pagano I., Dey A., Krausz T., Pass H.I., Yang H., Gaudino G. (2020). Biological Mechanisms and Clinical Significance of *BAP1* Mutations in Human Cancer. Cancer Discov..

[44] Popova T., Hebert L., Jacquemin V., Gad S., Caux-Moncoutier V., Dubois-D’enghien C., Richaudeau B., Renaudin X., Sellers J., Nicolas A. (2013). Germline BAP1 Mutations Predispose to Renal Cell Carcinomas. Am. J. Hum. Genet..

[45] Tan G., Xuan Z., Li Z., Huang S., Chen G., Wu Y., Chen X., Liang Z., Wu A. (2020). The critical role of BAP1 mutation in the prognosis and treatment selection of kidney renal clear cell carcinoma. Transl. Androl. Urol..

[46] Guo G., Gui Y., Gao S., Tang A., Hu X., Huang Y., Jia W., Li Z., He M., Sun L. (2011). Frequent mutations of genes encoding ubiquitin-mediated proteolysis pathway components in clear cell renal cell carcinoma. Nat. Genet..

[47] Joseph R.W., Kapur P., Serie D.J., Eckel-Passow J.E., Parasramka M., Ho T., Cheville J.C., Frenkel E., Rakheja D., Brugarolas J. (2013). Loss of BAP1 protein expression is an independent marker of poor prognosis in patients with low-risk clear cell renal cell carcinoma. Cancer.

[48] Kapur P., Peña-Llopis S., Christie A., Zhrebker L., Pavía-Jiménez A., Rathmell W.K., Xie X.-J., Brugarolas J. (2013). Effects on survival of BAP1 and PBRM1 mutations in sporadic clear-cell renal-cell carcinoma: A retrospective analysis with independent validation. Lancet Oncol..

[49] Zisman A., Pantuck A.J., Wieder J., Chao D.H., Dorey F., Said J.W., Dekernion J.B., Figlin R.A., Belldegrun A.S. (2002). Risk Group Assessment and Clinical Outcome Algorithm to Predict the Natural History of Patients With Surgically Resected Renal Cell Carcinoma. J. Clin. Oncol..

[50] Hakimi A.A., Ostrovnaya I., Reva B., Schultz N., Chen Y.-B., Gonen M., Liu H., Takeda S., Voss M.H., Tickoo S.K. (2013). Adverse Outcomes in Clear Cell Renal Cell Carcinoma with Mutations of 3p21 Epigenetic Regulators *BAP1* and *SETD2*: A Report by MSKCC and the KIRC TCGA Research Network. Clin. Cancer Res..

[51] Peña-Llopis S., Vega-Rubín-De-Celis S., Liao A., Leng N., Pavía-Jiménez A., Wang S., Yamasaki T., Zhrebker L., Sivanand S., Spence P. (2012). BAP1 loss defines a new class of renal cell carcinoma. Nat. Genet..

[52] Friedhoff J., Schneider F., Jurcic C., Endris V., Kirchner M., Sun A., Bolnavu I., Pohl L., Teroerde M., Kippenberger M. (2022). BAP1 and PTEN mutations shape the immunological landscape of clear cell renal cell carcinoma and reveal the intertumoral heterogeneity of T cell suppression: A proof-of-concept study. Cancer Immunol. Immunother..

[53] Liu K., Huang Y., Xu Y., Wang G., Cai S., Zhang X., Shi T. (2023). BAP1-related signature predicts benefits from immunotherapy over VEGFR/mTOR inhibitors in ccRCC: A retrospective analysis of JAVELIN Renal 101 and checkmate-009/010/025 trials. Cancer Immunol. Immunother..

[54] Yu M., Qian K., Wang G., Xiao Y., Zhu Y., Ju L. (2023). Histone methyltransferase SETD2: An epigenetic driver in clear cell renal cell carcinoma. Front. Oncol..

[55] Li F., Mao G., Tong D., Huang J., Gu L., Yang W., Li G.-M. (2013). The Histone Mark H3K36me3 Regulates Human DNA Mismatch Repair through Its Interaction with MutSα. Cell.

[56] Kanu N., Grönroos E., Martinez P., Burrell R.A., Goh X.Y., Bartkova J., Maya-Mendoza A., Mistrík M., Rowan A.J., Patel H. (2015). SETD2 loss-of-function promotes renal cancer branched evolution through replication stress and impaired DNA repair. Oncogene.

[57] Peña-Llopis S., Christie A., Xie X.-J., Brugarolas J. (2013). Cooperation and Antagonism among Cancer Genes: The Renal Cancer Paradigm. Cancer Res.

[58] Gerlinger M., Rowan A.J., Horswell S., Math M., Larkin J., Endesfelder D., Gronroos E., Martinez P., Matthews N., Stewart A. (2012). Intratumor heterogeneity and branched evolution revealed by multiregion sequencing. N. Engl. J. Med..

[59] Sato Y., Yoshizato T., Shiraishi Y., Maekawa S., Okuno Y., Kamura T., Shimamura T., Sato-Otsubo A., Nagae G., Suzuki H. (2013). Integrated molecular analysis of clear-cell renal cell carcinoma. Nat. Genet..

[60] Chen Y., Zheng X., Xiong J., Guan Y., Li Y., Gao X., Lin J., Fei Z., Chen L., Chen G. (2021). 79P SETD2 a potential tissue-agnostic predictive biomarker for ICIs in solid tumors. Ann Oncol..

[61] Lu M., Zhao B., Liu M., Wu L., Li Y., Zhai Y., Shen X. (2021). Pan-cancer analysis of SETD2 mutation and its association with the efficacy of immunotherapy. npj Precis. Oncol..

[62] Pearl L.H., Schierz A.C., Ward S.E., Al-Lazikani B., Pearl F.M. (2015). Therapeutic opportunities within the DNA damage response. Nat. Rev. Cancer.

[63] Pletcher J.P., Bhattacharjee S., Doan J.P., Wynn R., Sindhwani P., Nadiminty N., Petros F.G. (2021). The Emerging Role of Poly (ADP-Ribose) Polymerase Inhibitors as Effective Therapeutic Agents in Renal Cell Carcinoma. Front. Oncol..

[64] Rizvi N.A., Hellmann M.D., Snyder A., Kvistborg P., Makarov V., Havel J.J., Lee W., Yuan J., Wong P., Ho T.S. (2015). Cancer immunology. Mutational landscape determines sensitivity to PD-1 blockade in non–small cell lung cancer. Science.

[65] Le D.T., Uram J.N., Wang H., Bartlett B.R., Kemberling H., Eyring A.D., Skora A.D., Luber B.S., Azad N.S., Laheru D. (2015). PD-1 Blockade in Tumors with Mismatch-Repair Deficiency. N. Engl. J. Med..

[66] Teo M.Y., Seier K., Ostrovnaya I., Regazzi A.M., Kania B.E., Moran M.M., Cipolla C.K., Bluth M.J., Chaim J., Al-Ahmadie H. (2018). Alterations in DNA Damage Response and Repair Genes as Potential Marker of Clinical Benefit from PD-1/PD-L1 Blockade in Advanced Urothelial Cancers. J. Clin. Oncol..

[67] Ged Y., Chaim J., Knezevic A., Carlo M.I., Foster A., Feldman D.R., Teo M.Y., Riaz N., Lee C.-H., Patil S. (2019). Alterations in DNA damage repair (DDR) genes and outcomes to systemic therapy in 225 immune-oncology (IO) versus tyrosine kinase inhibitor (TKI) treated metastatic clear cell renal cell carcinoma (mccRCC) patients (pts). J. Clin. Oncol..

[68] Labriola M.K., Zhu J., Gupta R., McCall S., Jackson J., Kong E.F., White J.R., Cerqueira G., Gerding K., Simmons J.K. (2019). Characterization of tumor mutation burden, PD-L1 and DNA repair genes to assess relationship to immune checkpoint inhibitors response in metastatic renal cell carcinoma. J. Immunother. Cancer.

[69] Malumbres M., Barbacid M. (2009). Cell cycle, CDKs and cancer: A changing paradigm. Nat. Rev. Cancer.

[70] Satyanarayana A., Kaldis P. (2009). Mammalian cell-cycle regulation: Several Cdks, numerous cyclins and diverse compensatory mechanisms. Oncogene.

[71] Sherr C.J. (1995). D-type cyclins. Trends Biochem. Sci..

[72] Linehan W.M., Schmidt L.S., Crooks D.R., Wei D., Srinivasan R., Lang M., Ricketts C.J. (2019). The Metabolic Basis of Kidney Cancer. Cancer Discov..

[73] Clark D.J., Dhanasekaran S.M., Petralia F., Pan J., Song X., Hu Y., Leprevost F.d.V., Reva B., Lih T.-S.M., Chang H.-Y. (2020). Integrated Proteogenomic Characterization of Clear Cell Renal Cell Carcinoma. Cell.

[74] Logan J.E., Mostofizadeh N., Desai A.J., Von Euw E., Conklin D., Konkankit V., Hamidi H., Eckardt M., Anderson L., Chen H.-W. (2013). PD-0332991, a potent and selective inhibitor of cyclin-dependent kinase 4/6, demonstrates inhibition of proliferation in renal cell carcinoma at nanomolar concentrations and molecular markers predict for sensitivity. Anticancer Res..

[75] Chen D., Sun X., Zhang X., Cao J. (2020). Inhibition of the CDK4/6-Cyclin D-Rb Pathway by Ribociclib Augments Chemotherapy and Immunotherapy in Renal Cell Carcinoma. BioMed Res. Int..

[76] Alexandrov L.B., Nik-Zainal S., Wedge D.C., Aparicio S.A.J.R., Behjati S., Biankin A.V., Bignell G.R., Bolli N., Borg A., Børresen-Dale A.-L. (2013). Signatures of mutational processes in human cancer. Nature.

[77] Samstein R.M., Lee C.-H., Shoushtari A.N., Hellmann M.D., Shen R., Janjigian Y.Y., Barron D.A., Zehir A., Jordan E.J., Omuro A. (2019). Tumor mutational load predicts survival after immunotherapy across multiple cancer types. Nat. Genet..

[78] McGranahan N., Furness A.J.S., Rosenthal R., Ramskov S., Lyngaa R., Saini S.K., Jamal-Hanjani M., Wilson G.A., Birkbak N.J., Hiley C.T. (2016). Clonal neoantigens elicit T cell immunoreactivity and sensitivity to immune checkpoint blockade. Science.

[79] Turajlic S., Litchfield K., Xu H., Rosenthal R., McGranahan N., Reading J.L., Wong Y.N.S., Rowan A., Kanu N., Al Bakir M. (2017). Insertion-and-deletion-derived tumour-specific neoantigens and the immunogenic phenotype: A pan-cancer analysis. Lancet Oncol..

[80] Eckel-Passow J.E., Serie D.J., Cheville J.C., Ho T.H., Kapur P., Brugarolas J., Thompson R.H., Leibovich B.C., Kwon E.D., Joseph R.W. (2017). BAP1 and PBRM1 in metastatic clear cell renal cell carcinoma: Tumor heterogeneity and concordance with paired primary tumor. BMC Urol..

[81] Tabata M., Sato Y., Kogure Y., McClure M.B., Oshikawa-Kumade Y., Saito Y., Shingaki S., Ito Y., Yuasa M., Koya J. (2023). Inter- and intra-tumor heterogeneity of genetic and immune profiles in inherited renal cell carcinoma. Cell Rep..

[82] Li M., Li L., Zheng J., Li Z., Li S., Wang K., Chen X. (2023). Liquid biopsy at the frontier in renal cell carcinoma: Recent analysis of techniques and clinical application. Mol. Cancer.

[83] Aveta A., Cilio S., Contieri R., Spena G., Napolitano L., Manfredi C., Franco A., Crocerossa F., Cerrato C., Ferro M. (2023). Urinary MicroRNAs as Biomarkers of Urological Cancers: A Systematic Review. Int. J. Mol. Sci..

[84] Barkan E., Porta C., Rabinovici-Cohen S., Tibollo V., Quaglini S., Rizzo M. (2023). Artificial intelligence-based prediction of overall survival in metastatic renal cell carcinoma. Front. Oncol..

[85] Rizzo M., Cartenì G., Pappagallo G. (2014). We need both randomized trials and real-world data: The example of everolimus as second-line therapy for mRCC. Futur. Oncol..

